# Protocol for a systematic review of the use of qualitative comparative analysis for evaluative questions in public health research

**DOI:** 10.1186/s13643-019-1159-5

**Published:** 2019-11-01

**Authors:** Benjamin Hanckel, Mark Petticrew, James Thomas, Judith Green

**Affiliations:** 1 King’s College London, SUPHI, London, UK; 2 London School of Hygiene and Tropical Medicine, London, UK; 3 University College London, London, UK

**Keywords:** Systematic review, Qualitative comparative analysis, Public health, Complex health interventions

## Abstract

**Background:**

There is an increasing recognition that health intervention research requires methods and approaches that can engage with the complexity of systems, interventions, and the relations between systems and interventions. One approach which shows promise to this end is qualitative comparative analysis (QCA), which examines casual complexity across a medium to large number of cases (between 10 and 60+), whilst also being able to generalise across those cases. Increasingly, QCA is being adopted in public health intervention research. However, there is a limited understanding of how it is being adopted. This systematic review will address this gap, examining how it is being used to understand complex causation; for what settings, populations and interventions; and with which datasets to describe cases.

**Methods:**

We will include published and peer-reviewed studies of any public health intervention where the effects on population health, health equity, or intervention uptake are being evaluated. Electronic searches of PubMed, Scopus, Web of Science (incorporating Social Sciences Citation Index and Arts & Humanities Citation Index), Microsoft Academic, and Google Scholar will be performed. This will be supplemented with reference citation tracking and personal contact with experts to identify any additional published studies. Search results will be single screened, with machine learning used to check these results, acting as a ‘second screener’. Any disagreement will be resolved through discussion. Data will be extracted from full texts of eligible studies, which will be assessed against inclusion criteria, and synthesised narratively, using thematic synthesis methods.

**Discussion:**

This systematic review will provide an important map of the increasing use of QCA in public health intervention literature. This review will identify the current scope of research in this area, as well as assessing claims about the utility of the method for addressing complex causation in public health research. We will identify implications for better reporting of QCA methods in public health research and for reporting of case studies such that they can be used in future QCA studies.

**Systematic review registration:**

PROSPERO, CRD42019131910

## Background

Identifying the pathways that lead to the success of health interventions is crucial for public health research. However, assessing causal relationships between interventions and outcomes across contexts is challenging [1–3], as multiple interacting components come together in often complex ways [4–6] and ‘inevitable adaptations’ take place as health interventions get implemented within local contexts [3]. Recent work [7] has pointed to the limitations of using randomised control trials (RCTs) to examine complex health interventions, as they are often unrepresentative of everyday practices across contexts: there is increased input to achieve fidelity, participants in trials may be more committed to the intervention, and context plays an important role in shaping the intervention. Understanding complexity thus requires approaches that draw on real world case studies [2, 8] and can identify pathways to successful implementation of, and effective outcomes from, health interventions across diverse contexts.

Qualitative comparative analysis (QCA) is one approach that has been increasingly adopted in public health research and evaluation [8–12] to examine complexity. Developed by Charles Ragin [13, 14], QCA is a set-theoretic method, which ‘bridges’ quantitative (large-n statistical analyses) and qualitative (small-n case study) techniques to analyse complex causation across contexts [15]. A QCA analysis can involve a medium to large number of cases (between 10 and 60+), whilst also being able to generalise across those cases [16, 17]. QCA is often described as being suitable for ‘small-n’ scenarios and is less frequently used in large datasets, though some have used it with some success [18].

A QCA approach provides a tool for examining what conditions (alone or in combination with other conditions) are ‘necessary’ or ‘sufficient’ to produce some outcome [19]. QCA involves using Boolean algebra and formal logic to examine the presence or absence of conditions, and the effect on an outcome. QCA uses ‘truth tables’, which ‘list all the possible combinations of casual conditions and the empirical outcome associated with each configuration … to identify explicit connections between combinations of casual conditions and outcomes’ [19]. When first developed degree of membership was limited by nominal-scale variables, crisp sets, but has since been revised by Ragin to include ‘fuzzy sets’, which scale degree of membership from 0.0 to 1.0 [13, 19].

Warren et al. have argued that QCA offers an important contribution to mixed method public health evaluation methodologies [20]. Others have identified opportunities to use QCA for informing the translation of research/interventions into practice and policy [21, 22]. There is a growing body of scholarship in public health that does use QCA methods for these, and other, aims. However, to date, there has been no systematic review of the use of QCA in public health literature and how it is being adopted. This systematic review will address this gap, examining how it is being used in public health research, including the methods and cases used to explore complexity, the populations it is being applied to, and the types of intervention research that QCA has been used to examine. This is a timely point to assess the field, in order to identify strengths and weaknesses, gaps, and implications for how to better report QCA methods and the case studies on which future QCA studies might be based.

## Study aim

The primary aim of the review is to understand what is known about the purpose, role, and use of qualitative comparative analysis (QCA) in research on the uptake and public health effects of interventions.

The proposed systematic review will answer the following research questions:

1. How is QCA used for public health evaluation? What populations, settings, methods used in source case studies, unit/s and level of analysis (‘cases’), and ‘conditions’ (often referred to as ‘variables’ in public health) have been included in QCA studies?
2. What strengths and weaknesses have been identified by researchers who have used QCA to understand complex causation in public health evaluation research?
3. How is QCA used to understand the contexts in which interventions increase or decrease health inequities?
4. What are the existing gaps in, and strengths and weakness of, the QCA literature, and what implications do these have for future QCA studies for public health?

The study will also identify implications for better reporting of QCA methods in public health research and for reporting case studies in general.

## Methods/design

Our systematic review protocol was registered with the International Prospective Register of Systematic Reviews (PROSPERO) on 29 April 2019 (CRD42019131910). The protocol was prepared using the Preferred Reporting Items for Systematic Reviews and Meta-Analysis Protocols (PRISMA-P) 2015 statement [23].

### Eligibility criteria

Papers included in the systematic review will be published, be peer reviewed, and report on studies that have undertaken a qualitative comparative analysis (QCA) to examine casual complexity in the implementation, uptake, and/or effects of public health interventions. There will be no date restrictions.

#### Inclusion criteria

##### Types of participants

Studies will include any population (including national, geographical communities, population sub-groups). As the review will be examining the types of populations included in QCA studies, all study participants will be included. This includes youth and adult populations, their families, and public health professionals.

##### Types of interventions

QCA studies that incorporate case studies of any kind of public health interventions will be included. Public health interventions are defined here as those focused on the promotion or protection of health, or the prevention of ill health for populations. This is in contrast to clinical interventions, which focus on the prevention or treatment of illness in individuals [24]. This will include any interventions for where benefits to population health are the stated outcomes.

##### Types of outcome measures

To be included, QCA studies must identify an outcome (the phenomenon that the QCA is aiming to explain) that is relevant to public health, in which the effects on population health, health equity, or intervention uptake are being evaluated.

##### Types of studies

Papers reporting studies that use crisp and/or fuzzy sets will be included. QCA can be included as part of the analysis, or as the only tool used in analysing data.

Papers reporting both primary studies and systematic reviews which use QCA in their analysis will be included.

#### Exclusion criteria

Publications not in English.

Studies where no intervention is evaluated, including studies that use QCA to examine public health infrastructure (i.e. staff training for low-skilled health staff), or papers that report on prevalence of health issues (i.e. prevalence of child mortality).

### Search strategy

The search strategy will use the following phrases ‘Qualitative Comparative Analysis’ and ‘QCA’, which will be combined with keywords (i.e. ‘health’, ‘public health’, ‘intervention’, and ‘wellbeing’). Database searches will be conducted in PubMed, Scopus, Web of Science (incorporating Social Sciences Citation Index and Arts & Humanities Citation Index), Microsoft Academic, and Google Scholar. Study design must be QCA; no date limits will be imposed on the search. The search will be updated at the end of the review to ensure inclusion of all relevant studies. This will also be supplemented by reviewing reference lists of primary studies identified in the review, reviewing studies that cite these primary studies, as well as inviting experts to comment on whether the final list of studies omits any published studies known to them.

### Study selection and data collection

Data and records will be maintained by the lead investigator (BH) and stored on a shared secure platform for access by all investigators.

#### Selection process

Titles and abstracts identified by the bibliographic search and additional sources will be single screened against the criteria above, with machine learning employed to check these results, acting as a ‘second screener’ [25]. ‘EPPI-Reviewer’ Version 4 software, or equivalent machine learning software, will be used to screen titles and abstracts. After removing duplicates, each study will be categorised as ‘include’, ‘exclude’, or ‘unsure’. The full texts of articles categorised as ‘include’ and ‘unsure’ will be retrieved and then assessed against inclusion criteria by reviewers. Those categorised as ‘unsure’ and/or where there are disagreements will be resolved through discussion, and if necessary, a third reviewer will mediate any unresolved differences.

Data extracted will be tabulated, using a standardised form approved by all investigators to ensure consistency (see Table 1). This will include extracting the following information for each included paper: title, author, rationale for QCA, context (setting, case level of analysis, and population characteristics), health intervention, health outcome/s, methods of data generation pertaining to the cases included (qualitative and/or quantitative), findings, and study conclusions. The reviewer/s will also extract any strengths and limitations outlined in each paper. Study authors will be contacted regarding any uncertainties.

**Table 1:** Study data collection

Study characteristic	Specific items
Study identifiers	Title, authors, journal, publication year, county of publication, and sponsorship
Study aims	Rationale for using a QCA approach
Participants	Country/region, population(s), setting
Intervention characteristics	Public health intervention evaluated
Outcome	Phenomena explained by the QCA
Source case study methods	Methods of data generation pertaining to the cases included (qualitative and/or quantitative)
Other	Strengths and limitations of approach identified by authors

### Assessment of risk of bias

#### Quality assessment

The objective of this review is to examine how QCA is currently used in the public health literature. There are no established reporting criteria for quality, so no studies will be excluded on the basis of quality. However, there are suggestions as to how to best report QCA [15, 16]. We will utilise four key criteria from these recommendations to map current reporting of the method. These will be evidence of familiarity with cases, justification for selection of cases, availability of raw data matrix and ‘truth table’ outcomes, and solution formula reported.

## Data synthesis

Data will be synthesised narratively, using thematic synthesis methods [26–28]. Final studies included will, following a descriptive analysis, be grouped by outcome measures, as well as context of intervention (e.g. school). We will assess claims about the utility of the method for addressing complex causation in public health research by identifying how far the published QCA study was able to identify necessary and sufficient conditions for the selected outcome. We will analyse separately (as a sub sample) those studies with an outcome related to health equity or inequalities, to assess how far QCA has been used to date in this area.

## Discussion

QCA has been increasingly used in public health research, although the utility of the method for producing policy-relevant findings has been questioned [29]. The use of QCA in public health research therefore warrants further attention. We will map current use of the method, including identifying what types of intervention and setting have been addressed and what strengths and weaknesses researchers have identified in using QCA to address questions of complex causation. We will assess the extent to which current reporting in public health matches recommendations for best practice within the QCA research community, and the implications of these recommendations and current practice for the public health research community. As QCA relies on detailed knowledge of cases, we will also comment on the implications for public health researchers publishing stand-alone case studies, which might be used in future QCA studies. At present, there has been no systematic review of the use of QCA in public health literature and how it is being adopted; this systematic review will fill this gap, with the aim to inform, guide, and assess use of QCA in future complex health systems research.

There are several limitations to this study. Including only published studies from a rapidly emerging field may not be representative of the most recent applications of the method. In addition, we are only able to include those in English, which may underrepresent high-quality research from some regions.

## Data Availability

All relevant data are within the paper and its supporting information files.

## Abbreviations

QCA: Qualitative comparative analysis
RCT: Randomised control trial
